# Triptolide inhibits cell growth and GRP78 protein expression but induces cell apoptosis in original and radioresistant NPC cells

**DOI:** 10.18632/oncotarget.10412

**Published:** 2016-07-06

**Authors:** Chengmin Li, Bin Zhang, Wuwu Lv, Chen Lai, Zhikang Chen, Ran Wang, Xueying Long, Xueping Feng

**Affiliations:** ^1^ Institute of Medical Sciences, Xiangya Hospital, Central South University, Changsha, Hunan Province 410008, China; ^2^ Department of Histology and Embryology, Xiangya School of Medicine, Central South University, Changsha, Hunan Province 410008, China; ^3^ Department of General Surgery, Xiangya Hospital, Central South University, Changsha, Hunan Province 410008, China; ^4^ Department of Radiology, Xiangya Hospital, Central South University, Changsha, Hunan Province 410008, China

**Keywords:** nasopharyngeal carcinoma, CNE2, radioresistance, triptolide, GRP78

## Abstract

The radioresistance is the key factor to hamper curative effect and survival of nasopharyngeal carcinoma (NPC) patients. Nature triptolide (TPL) has been found to circumvent drug-resistant effect of cancer, but its effect on NPC radioresistance has been rarely studied. In the present study, the 10 Gy-resistant CNE2 subclones (CNE2-SR) were used as a NPC radioresistant model. The IC_50_ of TPL in CNE2 and CNE2-SR cells was measured by MTT assay, cell cycle was analyzed by flow cytometry, and protein expression was examined by western blot. Our data showed that TPL treatment decreased the percentage of viable cells, and IC50 value in CNE2 and CNE2-SR cells was 23.6 ± 1.41 nmol/L and 31.2 ± 1.16 nmol/L, respectively. Six Gy was a moderate dosage of X-ray for CNE2, and 25 nM TPL was close to IC50 value of CNE2 and CNE2-SR. Six Gy X-ray and/or 25 nM TPL significantly inhibited tumor growth in nude mice. Furthermore, 6 Gy X-ray and/or 25 nM TPL significantly inhibited cell growth and induced cell apoptosis and M/G2 phase arrest in CNE2 and CNE2-SR cells. Moreover, TPL treatment significantly inhibited the expression of GRP78 protein in CNE2 and CNE2-SR cells. These results suggest that TPL may serve as a potential radiosensitizer agent for NPC treatment.

## INTRODUCTION

Increasing incidence and mortality rate of cancers urge researchers to make great effort on searching for novel anti-cancer drugs or therapies. Many active compounds from natural herbs, such as Taxol, have been demonstrated to have antitumor activity and widely used in cancer therapy. Triptolide (TPL, molecular formula: C_20_H_24_O_6_) active components extracted from Chinese herb *Tripterygium wilfordii* Hook F (known as Lei Gong Teng or Thunder of God Vine) were usually used for rheumatoid arthritis therapy, but they also showed antitumor activity through inducing tumor cell apoptosis [1–3]. It has been found that TPL induces cell apoptosis via inhibiting HSP70 in pancreatic cancer cells [4]. Moreover, TPL treatment circumvents drug-resistant effect and enhances the anti-tumor effect of 5-fluorouracil in KB cells [5].

Nasopharyngeal carcinoma (NPC) is a kind of rare epithelial cancer, but it is one of the most prevalent malignancies in southern Chinese populations. However, the clinical outcome and prognosis of NPC remain unsatisfactory. Radioresistance is the biggest obstacle to achieve long-term survival. So it is urgent to screen some novel and effective drugs to treat radioresistant NPC. GRP78 is an ER chaperone protein and major regulator of the unfolded protein response (UPR) [6]. The response of GRP78 to drug-induced ER stress has been well established [6]. GRP78 may be an important biomarker protein for drug resistance.

The efficacy of TPL in NPC therapy and its molecular mechanisms have been poorly studied. In this study, we first examined the IC_50_ value of TPL in NPC CNE2 and CNE2-SR cells by MTT assay. Then, we further investigated that the effects of TPL on cell proliferation, cell cycle and the expression of GRP78 in both cells.

## RESULTS

### The radioresistant CNE2-SR from the irradiated CNE2 cells

**Figure 1:**
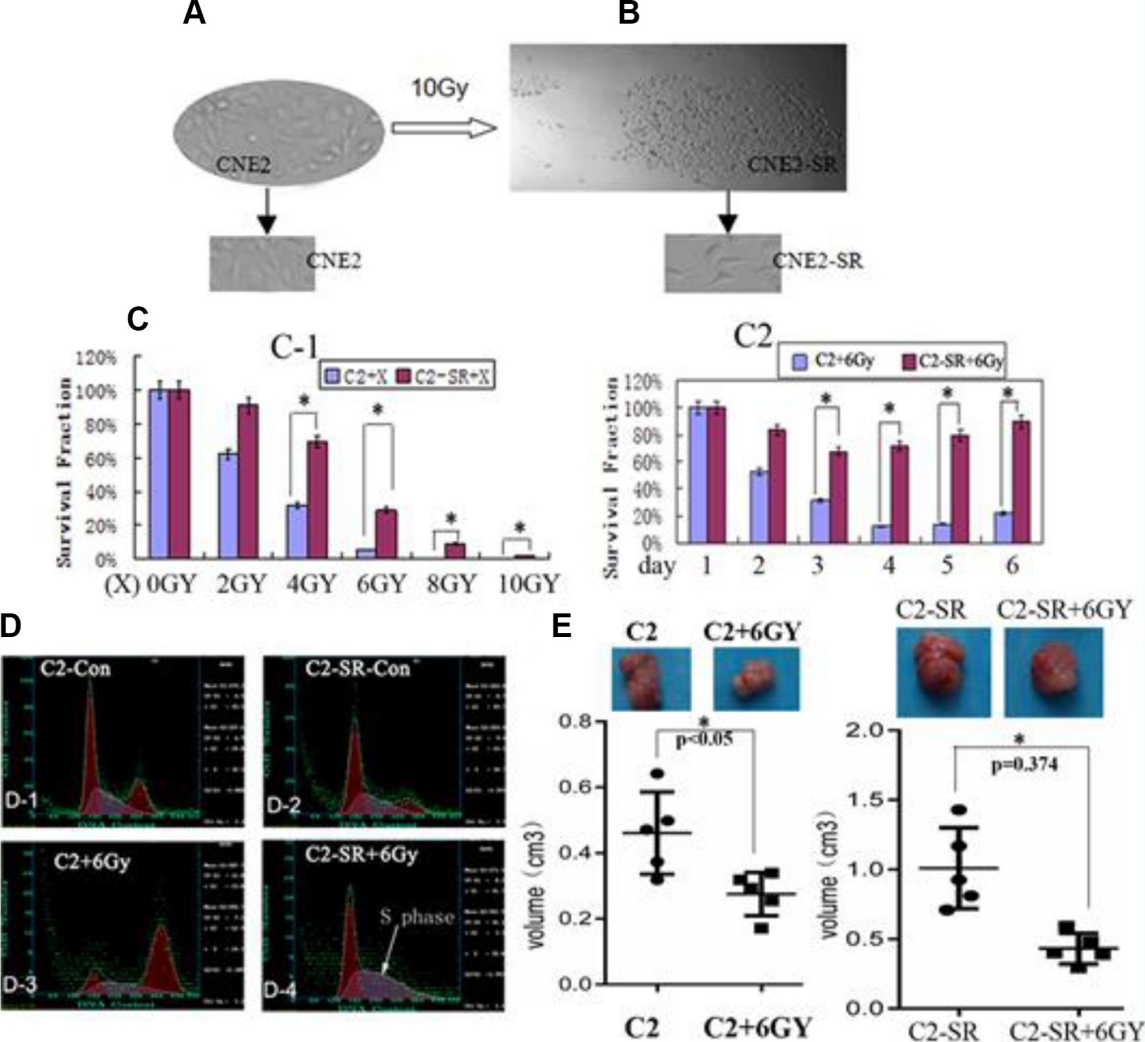
The radioresistant CNE2-SR subclones (**A**) NPC CNE2 cell line (CNE2); (**B**) CNE2-SR subclones (CNE2-SR) derived from the survival CNE2 cells by a large dosage of 10 Gy. (**C-1**) The survival fractions of CNE2-SR (C2-SR+X) were higher compared with CNE2 (C2 + X) after various X-ray (2-10 Gy); (**C-2**)The survival fractions of CNE2-SR (C2-SR + 6Gy) were higher compared with CNE2 (C2 + 6Gy) at various days (1–6 day) by 6 Gy. (**D**) Cell cycle analysis of CNE2 (C2+6Gy) and CNE2-SR (C2-SR + 6Gy) after 6 Gy X-ray for 48 h. (**E**) Measurement of tumor growth for CNE2 (C2 + 6Gy) and CNE2-SR (C2-SR + 6Gy) in nude mice (every group, *n* = 5) after treatment of 6 Gy.

The 10 Gy dose of X-ray radiation resulted in 95% of cell death in CNE2. After 15 days, the residual cells formed some clones (> 50 cells in each clone). The clones were more resistant than the parent CNE2 *in vitro* (Figure 1C–1D; Figure 3A) and *in vivo* (Figure 3D–3E). Then the clones were isolated and cultured as a radiation-resistant sample, CNE2-SR.

### TPL decreases cell viability and inhibits tumor growth

**Figure 2:**
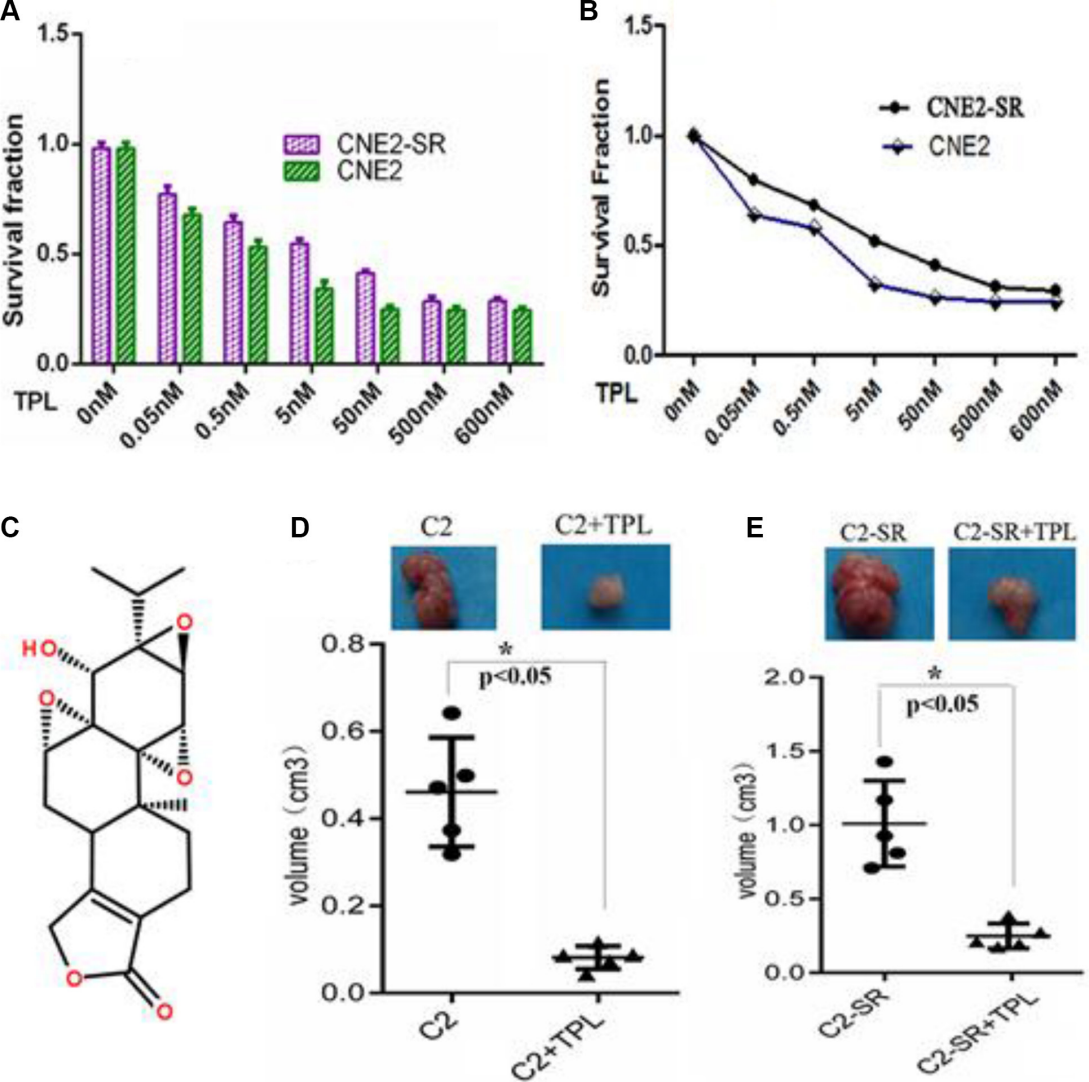
TPL inhibits cell proliferation in the CNE2-SR and CNE2 cells (**A**) Measurement of cell viability in CNE2 and CNE2-SR cells after treatment of various concentrations of TPL (0.05–500 nM) using MTT assay. The IC50 value of TPL in CNE2 and CNE2-SR cells was calculated. (**B**) Measurement of cell growth in CNE2 and CNE2-SR cells after treatment of various concentrations of TPL (0.05–500 nM) by counting cell number. The cell number in all treated groups was compared to that of untreated cells, and the survival of untreated cells was set as 1. (**C**) The structure of TPL molecule; (**D** and **E**) Measurement of tumor growth for CNE2 (C2) and CNE2-SR (C2- SR) in nude mice (every group, *n* = 5) after treatment of 25 nM TPL.

The viability in CNE2 and CNE2-SR cells was significantly decreased in a concentration-dependent manner after treatment with various doses (0.05–500 nM) of TPL (Figure 2A). Moreover, consistent with MTT assay, the number of viable cells was gradually decreased along with the increasing dose of TPL treatment (Figure 2B). There was no significant difference in the number of viable cells treated with various doses of TPL between CNE2 and CNE2-SR (*p* > 0.01) despite their different radiosensitivity (Figure 1A–1E). IC50 value in CNE2 and CNE2-SR cells was 23.6 ± 1.41 nmol/L and 31.2 ± 1.16 nmol/ L, respectively. The 25 nM TPL was close to IC50 value of CNE2 and CNE2-SR, and 25 nM TPL significantly inhibited tumor growth of CNE2 and CNE2-SR *in vivo* (Figure 2D and Figure 2E).

### Cell growth analysis in response to TPL and radiation

**Figure 3:**
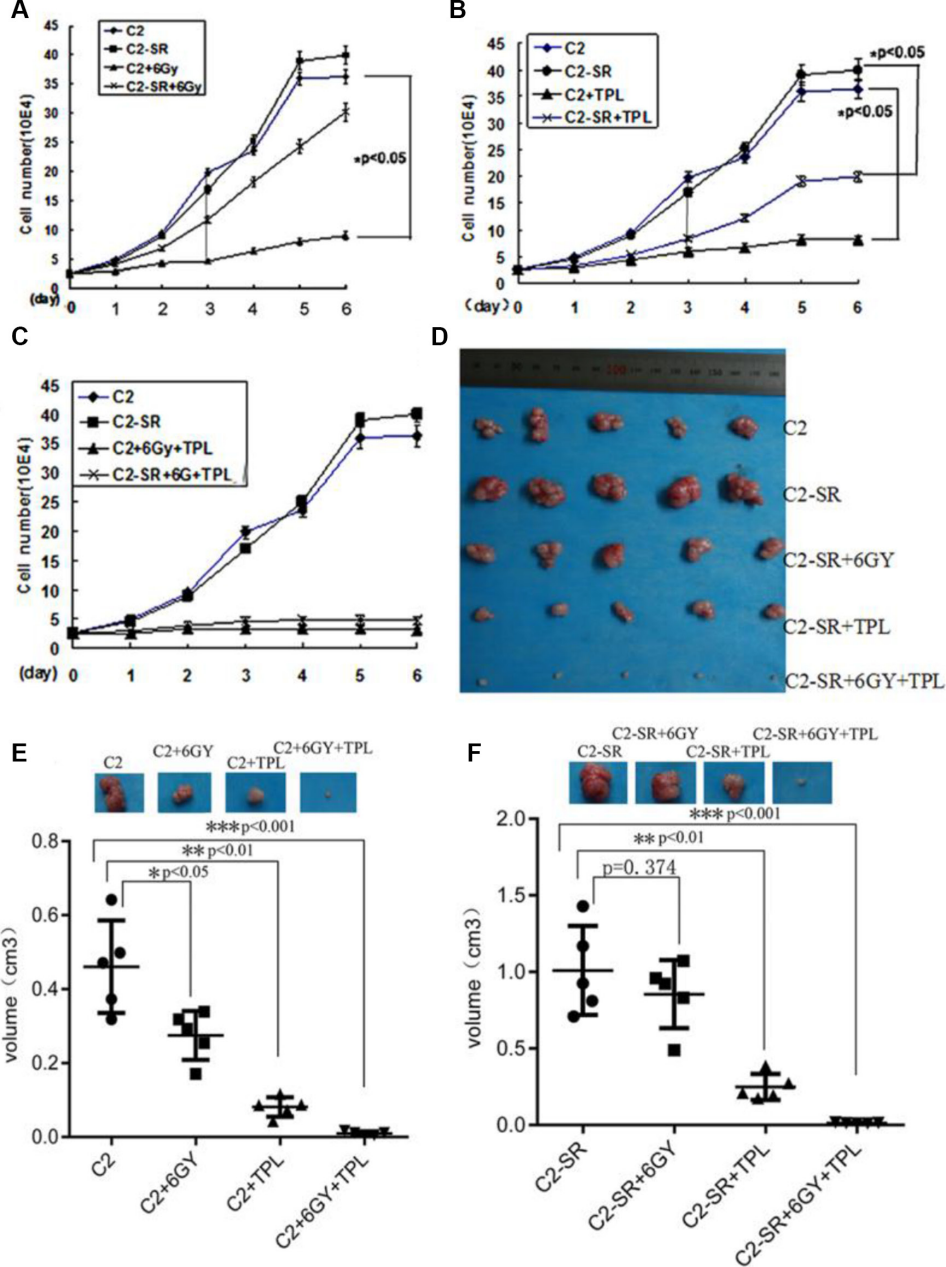
Effect of TPL and radiation on cell growth at different time points (**A**) Effect of 6 Gy X-ray (6 Gy) on cell growth in CNE2 (C2) and CNE2-SR (C2-SR) cells at different time points. (**B**) Effect of 25 nM TPL on cell growth in CNE2 and CNE-SR cells at different time points. (**C**) Effect of the combined treatments of 6 Gy X-ray and 25 nM TPL on cell growth in CNE2 and CNE2-SR cells at different time points. Cell growth was assessed by counting cell number. (**D**) The representative images of tumor. (**E**) and (**F**) the analysis of tumor growth for CNE2 (C2) and CNE2-SR (C2-SR) after treatment of X-ray and/or 25 nM TPL in nude mice (every group, *n* = 5).

Six Gy was a moderate dosage of X-ray for CNE2, and 25 nM TPL was close to IC50 value in CNE2 and CNE2-SR. The proliferation of CNE2 and CNE2-SR cells was monitored at various time intervals after treatment with 6 Gy X-ray and/or 25 nM TPL. As shown in the Figure 3, both CNE2 and CNE2-SR cells were sensitive to 25 nM TPL treatments (Figure 3B), but CNE2-SR cells were more resistant to 6 Gy X-ray compared with CNE2 cells (Figure 3A). Moreover, the combined treatments of 6 Gy X-ray and 25 nM TPL completely inhibited cell proliferation in both cell lines *in vitro* (Figure 3C) and *in vivo* (Figure 3D–3E).

### TPL treatment enhances the anti-tumor effect of X-ray *in vivo*

To confirm the radiosensitizer effect of TPL to CNE2-SR cells, TPL was tested with radiation-resistant CNE2-SR *in vivo*. The radiosensitizer effects of TPL on NPC radioresistance were tested in nude mice. The combined treatments of 6 Gy X-ray or/and 25 nM TPL significantly inhibited tumor growth of the radioresistant CNE2-SR in nude mice (Figure 3D and 3E). TPL treatment enhanced the anti-tumor effect of X-ray, and circumvented the resistant effect of X-ray in the nude mice transplanted with CNE2-SR cells (Figure 3E).

### TPL enhances the radiosensitivity of cells and changes cell cycle transition

**Figure 4:**
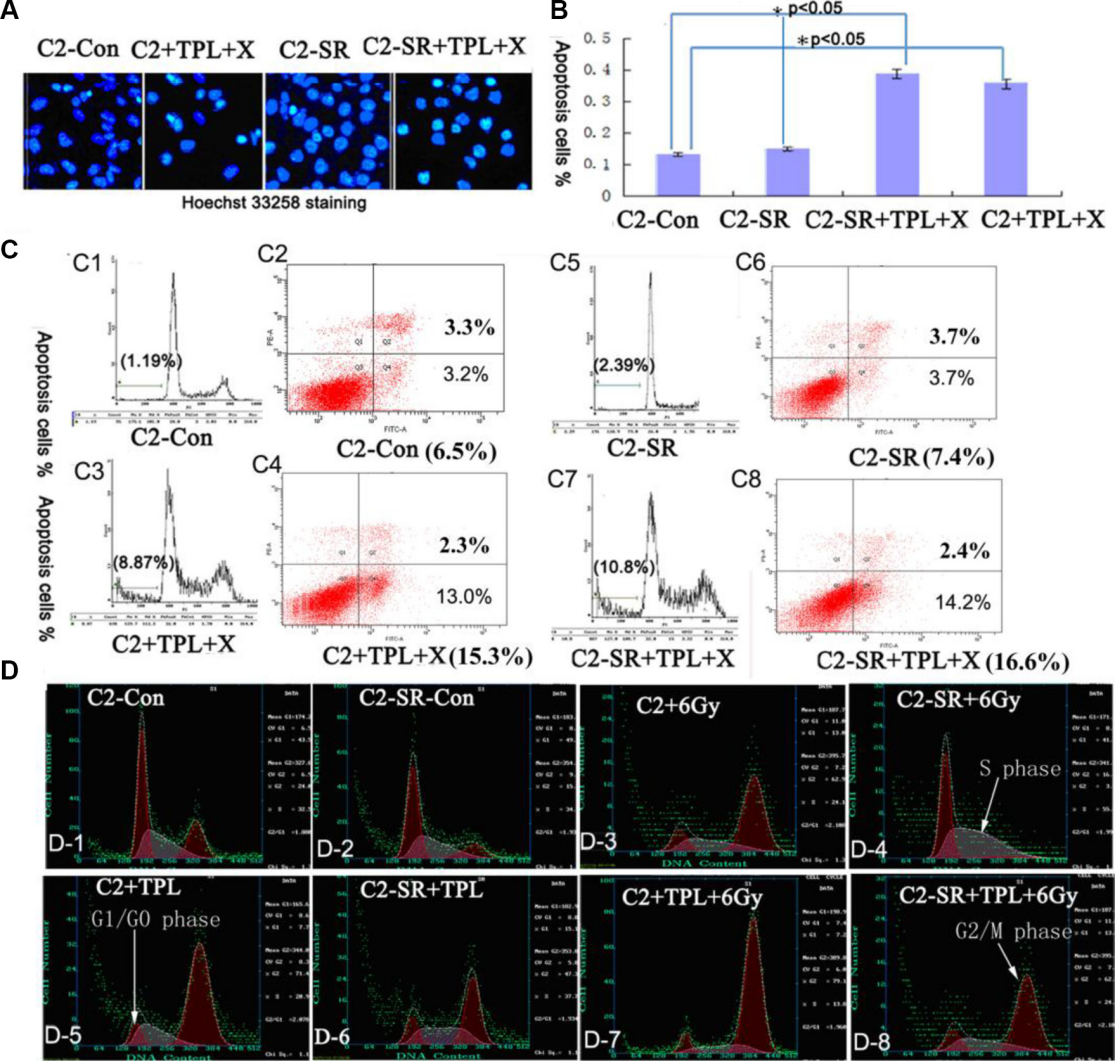
TPL induces cell apoptosis and M/G2 phase arrest (**A**) Assay of cell apoptosis by Hoechst 33258. Both CNE2 (C2) and CNE2-SR (C2-SR) cells were cultured in 6-well culture plates and treated with 6 Gy X-ray and 25 nM TPL for 48 h. (**B**). Statistical analysis cell apoptosis based on Hoechst 33258 staining from three independent experiments (*n* = 3). The data are presented as mean ± standard deviation (SD). *p < 0.05. (**C**) Measurement of cell apoptosis by flow cytometry after Annexin V-FITC/propidium iodide (PI). (**D**) Cell cycle analysis of CNE2 and CNE2-SR cells after 6 Gy X-ray and/or 25 nM TPL for 48 h.

**Figure 5:**
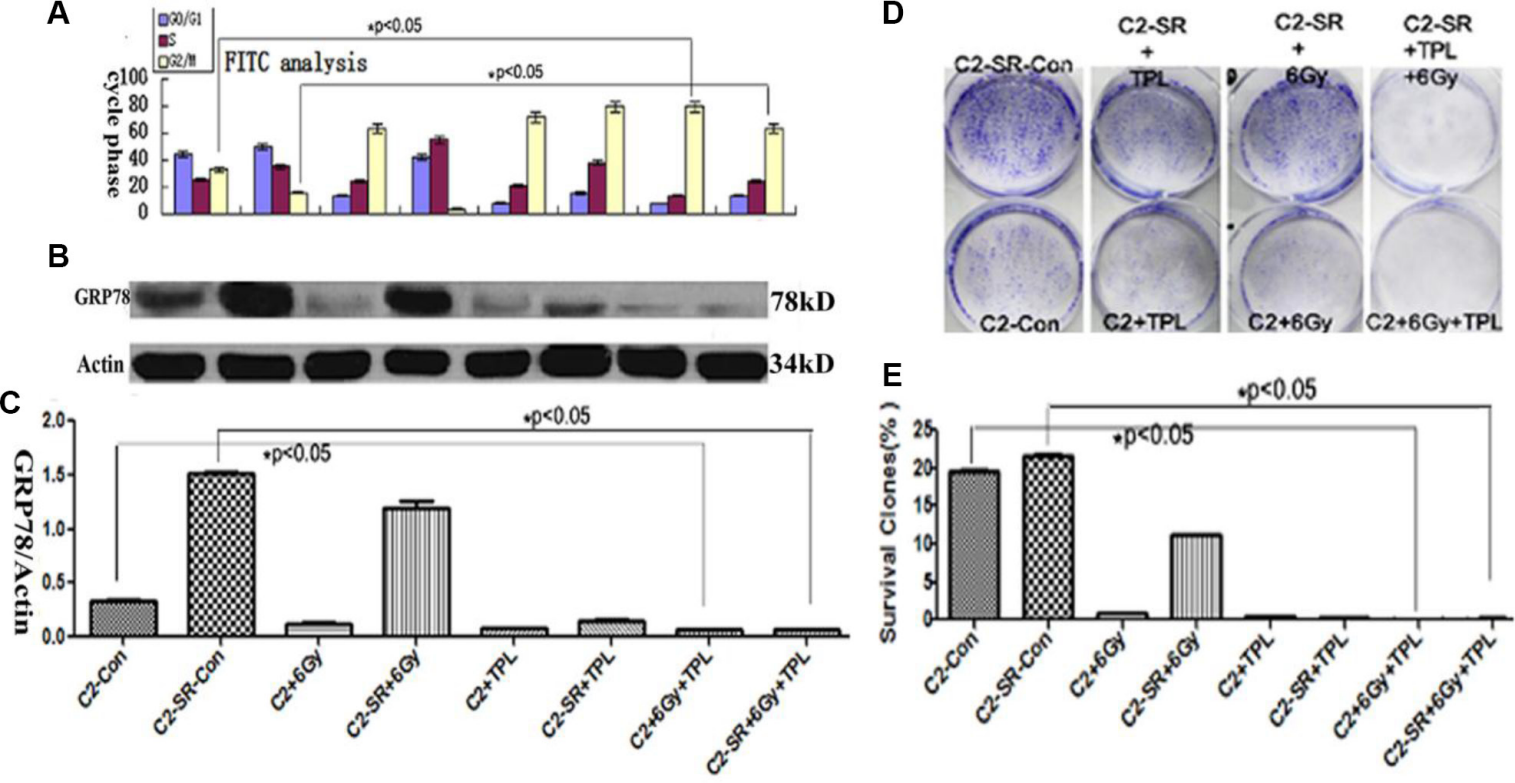
TPL inhibits the expression of GRP78 protein and colony formation capacity (**A**) Cell cycle analysis of CNE2 and CNE2-SR cells after 6 Gy X-ray and/or 25 nM TPL. (**B**) Effect of 6 Gy X-ray and/or 25 nM TPL on GRP78 expression in CNE2 (C2) and CNE2-SR (C2-SR) cells and (**C**) Statistical analysis of GRP78 protein expression from three independent experiments (*n* = 3). The data are presented as mean ± SD, *P < 0.05. (**D**) Effect of 6 Gy X-ray and/or 25 nM TPL on colony assay in CNE2 (C2) and CNE2-SR (C2-SR) cells. (**E**) Statistical analysis of colony formation in CNE2 and CNE2-SR cells. The data are presented as mean ± SD. *p < 0.05.

We examined the effect of combined treatments of 6 Gy X-ray and 25 nM TPL on cell apoptosis and cell cycle analysis by the staining of Hoechst 33258 and Annexin V-FITC/p*ropidium iodide (PI)*. As shown in the Figure 4, the combined treatments of 6 Gy X-ray and 25 nM TPL significantly induced cell apoptosis in CNE2 and CNE2-SR cells (Figure 4A–4C). We further found that the majority of untreated CNE2 and CNE2-SR cells were in G0/G1 phase(Figure 4D_1–2_ and Figure 5A). The treatments of 25 nM TPL or/and 6 Gy significantly promoted cell cycle transition from G0/G1 to G2/M phase (Figure 4D_3,5,7_; Figure 5A) in CNE2 cells, but this effect was not observed in CNE2-SR cells treated with 6Gy X-ray (Figure 4D_4_; Figure 5A). However, the treatment of 6 Gy X-ray in CNE2-SR significantly promoted the transition from G2/M to S phase (Figure 4D_4_; Figure 5A). Moreover, the combined treatments of 6 Gy X-ray and 25 nM TPL in CNE2-SR cells further promoted cell cycle transition from G0/G1 to G2/M phase again (Figure 4D_8_).

### TPL inhibits GRP78 protein expression and colony formation

Since GRP78 is one of important biomarkers for drug-induced ER stress [6], here we examined the expression of GRP78 protein. As shown in the Figure 5, the expression of GRP78 protein in CNE2-SR cells was significantly higher than that in CNE2 cells, while 25 nM TPL but not 6 Gy X-ray significantly inhibited the expression of GRP78 protein after individual or combined treatments (Figure 5B and 5C). Interestingly, the level of GRP78 protein was negatively correlated with the proportion of G2/M phase cells (Figure 5A–5C). Moreover, we examined the colony formation capacity after 25 mM TPL and/or 6 Gy X-ray treatments (Figure 5D and 5E). Both 6 Gy X-ray and 25 nM TPL treatments significantly inhibited colony formation in CNE2 and CNE2-SR cells (Figure 5D and 5E).

## DISCUSSION

The standard combination of radiotherapy and chemotherapy has improved the efficiency in NPC patients, but radioresistance is still the common causes of biggest obstacle in NPC treatment [7–9]. The survival of irradiated NPC cells is the most common cause for the relapse and metastasis of NPC. In the present study, we established radioresistant CNE2-SR subclones from CNE2 cells by 10 Gy X-ray radiation, which were more resistant to 6 Gy X-ray radiation compared with original CNE2 cells. TPL is a natural drug with anticancer efficacy, and has been used as a good alternative in many cancer treatments. Early in 1972, Kupchan *et al*. first reported the antileukemic effect of TPL [10]. The combination of TPL and X-ray showed synergistic effects in preclinical animal models [11]. Jacobson *et al*. [12] found that TPL inhibits pancreatic cancer cell proliferation through reducing the expression of heat shock protein 70. However, the efficacy of TPL in NPC therapy has not yet been addressed.

Our data showed that TPL decreased the percentage of viable cells in the radioresistant CNE2-SR cells. The combination of TPL and X-ray showed synergistic effects and circumvented the resistant effect of X-ray in the nude mice transplanted with CNE2-SR cells. It has been demonstrated that the cells at G2/M phase are most sensitive to radiation, and G2/M phase arrest is one of the major reasons of cell death caused by anti-tumor drugs and radiosensitive reagents [13]. Significant elevation of GRP78 expression in cancer cells is linked to a radioresistant cell type [6–7]. GRP78 is known to induce anti-apoptosis, chemoresistance and radioresistance. GRP78 has been considered as a potential biomarker for therapy-resistance in multiple carcinomas [14–15]. Li *et al*. found that GRP78 enhances the glutamine metabolism and supports cell survival from glucose deficiency by activating the β-catenin signaling [14], while GRP78 inhibitor OSU-03012/sildenafil could kill brain cancer stem cells [15]. This evidence suggests that the down-regulation of GRP78 may inhibit cell survival. Here we observed that the combined treatments of 6 Gy X-ray and 25 nM TPL could promote cell cycle transition from G0/G1 to G2/M phase in the radioresistant CNE2-SR cells. Moreover, the G2 /M phase arrest and cell apoptosis in CNE2-SR cells accompanied the downregulation of GRP78 protein expression. Hence, we speculate that the elevated GRP78 protein may support cell survival and enhance radioresistance to 6 Gy X-ray radidation in CNE2-SR cells.

TPL has been found to sensitize cancer cells to apoptosis [16–18]. Moreover, a recent study reported that the expression of GRP78 in pancreatic cancer cell lines is downregulated by TPL, leading to cell apoptosis and autophagy [19]. TPL may provide new insight and knowledge for future studies in NPC radioresistant field. Radioresistance is mostly associated with numerous molecular aberrations that characterize glioblastoma, such as enhanced DNA damage repair (DDR) and activation of pro-survival signaling pathways [21]. GRP78 is a critical pro-survival chaperone involved in ER stress linked to the changes in ROS balance secondary to irradiation [22]. Consistently, our results suggest that TPL may trigger apoptosis and induce G2/M phase arrest via inhibiting GRP78. Thus, TPL may serve as a potential chemotherapeutic agent and new radiosensitizer for NPC treatment.

## MATERIALS AND METHODS

### The survival subclones of CNE2 cells to irradiation

CNE2 cells were seeded at a density of 1.0 × 10^5^ cells per 6 cm plate and cultured in RPMI-1640 medium (Gibco BRL) containing 10% heat-inactivated fetal bovine serum (FBS, HyClone) and 1% antibiotics at 37°C in a humidified 5% CO_2_ incubator. After 24 h culture, the cells were exposed to sublethal 10 Gy dose (300 cGy/min) of X-ray at room temperature using a linear accelerator (2100EX, Varian). Fifteen days after treatment, the residual CNE2 cells were cultured and named for CNE2-SR (CNE2 Survival-Residual subclones). CNE2 cells irradiated with sham were used as a control.

### Cell viability analysis by MTT assay

To evaluate the anticancer effect of TPL on NPC cell lines, IC50 of TPL in CNE2 and CNE2-SR cells was examined by 3-(4, 5-dimethylthiazol-2-yl)-2, 5-diphenyltetrazolium bromide (MTT) assay. Cells were cultured at 5.0 × 10^3^/well in 96-well plates for 24 h, then treated with 0.01 mM PBS (control samples) and various doses (0.05 nM, 0.50 nM, 5 nM, 50 nM, 500 nM and 600 nM, diluted in culture medium.) of TPL for 48 h. 20 μl of MTT dye (3 g/L in PBS; Sigma-Aldrich) were added to each well and incubated for 4 h. The absorbance of each well was read at 630 nm on an EL310 Microplate Autoreader (Bio-Tek Instruments). Cell viability was calculated by comparison of the A630 readings to the control absorbance. Three independent experiments were conducted. IC50 value of TPL in CNE2 and CNE2-SR cells was calculated.

### Cell growth analysis by cell counting

CNE2 and CNE2-SR cells were plated at a density of 4 × 10^4^ cells/well in triplicate in a 6-well plate and exposed to 25 nM of TPL and/or 6 Gy of X-ray. Cell growth was monitored by counting cell number at various time intervals. Three independent experiments performed.

### Cell apoptosis and cell cycle analysis by flow cytometry

2.5 × 10^6^ cells were treated with TPL (25 nM) and/or X-ray (6 Gy) and harvested after 48 h treatment. Cell pellets were fixed with ice-cold 70% ethanol in PBS at −20°C for 1 h and then centrifuged at 1,500 rpm for 5 min. The pellet was incubated with 0.5% Triton X-100 (Sigma) and 0.05% RNase (Sigma) in 1 mL PBS at 37°C for 30 min and then centrifuged at 1,500 rpm for 5 min. For cell cycle analysis, cell pellets were resuspended in 1 mL PBS containing 40 μg/mL propidium iodide (PI, Sigma) and incubated for 30 min in dark at room temperature. For cell apoptosis, cells were washed with 1×PBS (4°C), incubated with 300 μL 1×Binding Buffer containing 5 μL Annexin V-FITC and 5 μL with PI for 30 min in dark at room temperature. Samples were immediately analyzed by a FAC-Scan flow cytometry (Becton–Dickinson). The distribution of cell cycle was analyzed using Flow Jo, CellQuest Pro and the ModiFit software. Three independent experiments were performed.

### Hoechst 33258 staining of cells

Cells were grown on coverslips in 6-well plate. The morphology and apoptosis of cells were examined by staining with cell-permeable DNA dye Hoechst 33258 (Sigma). Briefly, cells were fixed with ice-cold 70% ethanol in PBS for 30 min after exposure to TPL (25 nM) and/or X-ray (6 Gy) for 48 h, then stained with 5 μg/mL cell-permeable DNA dye Hoechst 33258 (Sigma) dissolved in Hanks' buffer for 10 min in the dark. Cells with homogeneously stained nuclei were considered to be viable, whereas the presence of chromatin condensation and/or fragmentation was indicative of apoptosis. To calculate the percentage of apoptotic cells, at least 200 cells in three different microscopic fields were evaluated.

### Western blot analysis

CNE2 and CNE2-SR cells were seeded at a density of 8 × 10^4^ cells/well in a 6-well plate and cultured in the presence of TPL (25 nM) and/or X-ray (6 Gy) for 48 h. Western blot was carried out as previously described [7]. Briefly, 50 μg of cell lysates were separated by 10% SDS-PAGE and transferred onto polyvinylidene difluoride membrane. Blots were blocked with 5% nonfat milk for 2 h at room temperature and then incubated with polyclonal goat anti-GRP78 antibody (1: 2,000 dilution, Santa Cruz Biotechnology, Santa Cruz, CA) at 4°C overnight. The membranes were washed and incubated with horseradish peroxidase-conjugated secondary antibody (1: 2000, Santa Cruz Biotechnology). As a loading control, β-actin was detected simultaneously using monoclonal mouse anti–β-actin antibody (1: 3,000 dilution, Sigma). Visualization was performed with an Amersham-enhanced chemiluminescence system. The intensity of bands was quantified by software. All experiments were repeated three times.

### Colony formation assay

Radioresistance of cells was measured by clonogenic survival assay following exposure to irradiation. The clonogenic survival assay was carried out as described previously [7, 20]. Briefly, cells were plated in six-well culture plates and were exposed to 6 Gy X-ray. After irradiation, the cells were cultured for 15 days and the number of surviving colonies (defined as a colony with > 50 cells) was counted. After staining with crystal violet, cell colonies were counted under a microscope. The survival fraction was calculated based on the formula: number of colonies counted/(number of cells plated × plating efficiency). Three independent experiments were performed.

### The radiosensitizer effects of TPL in Xenograft mouse model

To evaluate the pharmacodynamics of TPL, we compared the antitumor efficiency of TPL or/and X-ray in xenograft mouse model of CNE2 and CNE2-SR *in vivo*. Three week-old nude mice were subcutaneously inoculated with 1 × 10^7^ CNE2/CNE2-SR cells diluted with 1640 medium. The tumors were treated in the presence of TPL (25 nM) and/or X-ray (6 Gy) after tumor formation. The body weight and tumor volume were monitored and calculated every 3 days for 30 days. The tumor volumes were calculated by the equation V (mm^3^) = a × b^2^/2, where “a” is the largest diameter and “b” is the perpendicular diameter.

### Statistical analysis

Statistical analyses were performed using SPSS 13.0 and Graphpad Prism 5.0. The difference in GRP78 expression between CNE2 and CNE2-SR cells was analyzed using *T* test. *P* < 0.05 was considered statistically significant.
